# KRT8 and KRT19, associated with EMT, are hypomethylated and overexpressed in lung adenocarcinoma and link to unfavorable prognosis

**DOI:** 10.1042/BSR20193468

**Published:** 2020-07-03

**Authors:** Wenlong Wang, Junhong He, Hongda Lu, Qingzhi Kong, Shengyou Lin

**Affiliations:** 1 Intensive Care Unit, Hangzhou Hospital of Traditional Chinese Medicine, Zhejiang Province, Hangzhou 310007, China; 2 Clinical College of Traditional Chinese Medicine, Hubei University of Traditional Chinese, Hubei Wuhan 430065, China; 3 Department of Emergency, Yiwu Central Hospital, Zhejiang Province, Yiwu 322000, China; 4 The Central Hospital of Wuhan, Tongji Medical College, Huazhong University of Science and Technology, Hubei Province, Wuhan 430014, China; 5 Department of Oncology, Hangzhou Hospital of Traditional Chinese Medicine, Zhejiang Province, Hangzhou 310007, China

**Keywords:** bioinformatic analysis, epithelial-to-mesenchymal transition (EMT), Lung adenocarcinoma (LUAD), mesenchymal-to-epithelial transition (MET), methylation

## Abstract

**Background:** Lung adenocarcinoma (LUAD) is the most common histological type of lung cancer. To date, the prognosis of patients with LUAD remains dismal. **Methods:** Three datasets were downloaded from the GEO database. Differentially expressed genes (DEGs) were obtained. FunRich was used to perform pathway enrichment analysis. Protein–protein interaction (PPI) networks were established and hub genes were obtained by Cytoscape software. GEPIA was utilized to conduct correlation and survival analysis. Upstream miRNAs of DEGs were predicted via miRNet database, and methylation status of promoters of DEGs was determined through UALCAN database. **Results:** A total of 375 DEGs, including 105 and 270 up-regulated and down-regulated genes in LUAD, were commonly appeared in three datasets. These DEGs were significantly enriched in mesenchymal-to-epithelial transition (MET) and epithelial-to-mesenchymal transition (EMT). About 8 up-regulated and 5 down-regulated DEGs were commonly appeared in EMT/MET-related gene set and the top 50 hub gene set. Among the 13 genes, increased expression of KRT8 and KRT19 indicated unfavorable prognosis whereas high expression of DCN and CXCL12 suggested favorable prognosis in LUAD. Correlation analysis showed that KRT8 (DCN) expression was linked to KRT19 (CXCL12) expression. Further analysis displayed that KRT8 and KRT19 could jointly forecast poor prognosis in LUAD. About 42 and 2 potential miRNAs were predicted to target KRT8 and KRT19, respectively. Moreover, methylation level analysis demonstrated that KRT8 and KRT19 were significantly hypomethylated in LUAD compared with normal controls. **Conclusions:** All these findings suggest that KRT8 and KRT19 are hypomethylated and overexpressed in LUAD and associated with unfavorable prognosis.

## Background

Lung cancer is the most prevalent cancer and the deadliest malignancy worldwide, which has led to a number of important public health problems [1]. In the United States, lung cancer is the second most common incidence cancer and the leading cause of cancer death, with the estimated new cases of lung cancer being 22,300 and cancer deaths being 56,000 [2]. In China, an estimated 4,292,000 new cases and 2,814,000 cancer deaths occurred in 2015, with lung cancer being the most commonly diagnosed cancer and the leading cause of cancer death [3]. Lung adenocarcinoma (LUAD), the most common histological type of lung cancer, has been a serious human health problem. Despite substantial advancements in therapeutic regimens including surgery, chemotherapy, radiotherapy and epidermal growth factor receptor tyrosine kinase inhibitor therapy, the prognosis of LUAD is still dismal, with 5-year survival rate less than 20% [2,4]. Moreover, the morbidity of LUAD has been increasing year by year. Therefore, it is urgent need to seek more effective drug candidates and promising diagnostic and prognostic biomarkers for LUAD.

The Gene Expression Omnibus (GEO) database is an international public repository, which provides a flexible and open design that can facilitate submission, storage and retrieval of heterogeneous data sets from high-throughput gene expression and genomic hybridization experiments [5,6]. Numerous studies have suggested that many potential tumor biomarkers can be identified by deep-mining the data in the GEO database [7,8].

To the best of our knowledge, the molecular pathological mechanisms of LUAD remain largely unknown. The objective of this work is to find the key genes and their potential regulatory mechanisms in LUAD. In the present study, three gene expression microarray datasets from the GEO database were first analyzed to obtain differentially expressed genes (DEGs) using the online analytic tool, GEO2R. Next, we conducted pathway enrichment analysis for the DEGs that were commonly appeared in all the three datasets, and have successfully identified two significant pathways: epithelial-to-mesenchymal transition (EMT) and mesenchymal-to-epithelial transition (MET). Subsequently, protein–protein interaction networks were established and hub genes were identified. Further survival analysis for these potential genes revealed that two up-regulated and two down-regulated genes possessed significant prognostic values in patients with LUAD. Next, we assessed the correlation and joint prognostic values of the key genes. Finally, potential upstream miRNAs and methylation status of the key genes were also predicted and determined. Findings from this study may provide critical clues for seeking and developing promising diagnostic, therapeutic and prognostic biomarkers in LUAD.

## Methods

### Gene expression microarray

In the discovery step, we included datasets that compared the gene expression in LUAD tissues with normal lung tissues. Only datasets containing more than 40 clinical samples (normal samples more than 20 and cancer samples more than 20) were included. Subsequently, the titles and abstracts of these datasets were screened, and the full information of the datasets of interest were further evaluated. Finally, only three datasets (GSE7670, GSE10072 and GSE32863) met these criteria and were selected for further analysis. Three datasets, the dataset GSE7670 based on the platform of GPL96 (Affymetrix Human Genome U133A Array) containing 27 LUAD samples and 27 normal samples, the dataset GSE10072 based on the platform of GPL96 (Affymetrix Human Genome U133A Array) containing 58 LUAD samples and 49 normal samples, the dataset GSE32863 based on the platform of GPL6884 (Illumina HumanWG-6 v3.0 expression beadchip) containing 58 LUAD tissues and 58 matched adjacent normal lung tissues, were downloaded from the National Center for Biotechnology Information (NCBI) GEO database (https://www.ncbi.nlm.nih.gov/geo).

### Screening of DEGs

We performed the comparison on the two groups of samples (LUAD versus normal lung tissues) in each selected GEO dataset to obtain the DEGs by using the online analytic tool GEO2R (https://www.ncbi.nlm.nih.gov/geo/geo2r), which was provided by the GEO database. Adjusted *P*-value (adj. *P*-value) < 0.05 and |log_2_FC| > 1 were set as cut-off criteria. The adj. *P*-value from the Benjamini–Hochberg method corrected the false positive results. The Venn diagram was drawn using online tool VENNY 2.1.0 (http://bioinfogp.cnb.csic.es/tools/venny/index.html). Among these DEGs from the three datasets, only DEGs commonly appeared in all three datasets (intersection set) were considered as the significant DEGs and were selected for subsequent analysis.

### Pathway enrichment analysis

FunRich, a stand-alone software tool used mainly for functional enrichment and interaction network analysis of genes and proteins, was utilized to perform pathway enrichment analysis for these significant DEGs [9]. The top ten enriched pathways were generated and displayed in the software. *P*-value < 0.05 was considered as statistically significant.

### Protein–protein interaction network establishment and analysis

Protein–protein interaction (PPI) networks of the DEGs were constructed using online database resource Search Tool for the Retrieval of Interacting Genes (STRING) (http://string-db.org/) [10,11]. The DEGs were entered into the database and high-resolution bitmaps were downloaded (each pair of interactors in the DEGs with combined confidence score > = 0.4). To identify hub genes in the PPI networks, CytoHubba of Cytoscape software (version 3.6.1) was introduced to analyze the degree of connectivity as previously reported [12–14]. According to degree, the top 50 hub genes were screened out, and the PPI networks of top 10 hub genes were displayed in Cytoscape software.

### GEPIA database analysis

Gene Expression Profiling Interactive Analysis (GEPIA) (http://gepia.cancer-pku.cn/detail.php), a web-based tool to deliver fast and customizable functionalities based on The Cancer Genome Atlas (TCGA) and Genotype-Tissue Expression (GTEx) data, was used to determine gene expression levels in LUAD compared with normal controls [15]. *P*-value < 0.05 and |log_2_FC| > 1 were regarded as statistically significant. GEPIA database was utilized to conduct Spearman correlation analysis for these identified key genes. *P*-value < 0.05 was considered as statistically significant. Besides, GEPIA database was also employed to jointly predicting prognostic values of KRT8 and KRT19 or DCN and CXCL12 in LUAD. Logrank *P*-value < 0.05 was considered as statistically significant.

### Survival analysis

The prognostic roles of the mRNA expression of 13 potential genes in patients with LUAD were evaluated using the Kaplan–Meier Plotter (http://www.kmplot.com), which is an online database including gene expression data and clinical data, partially from the TCGA database [16]. LUAD (*n*=513) mRNA RNA-seq data from ‘Pan-cancer’ item was selected. These genes were entered into the database, and the hazard ratio (HR) with 95% confidence interval and logrank *P*-value were calculated and displayed. Logrank *P*-value < 0.05 was considered as significant.

### miRNet database analysis

miRNet, an integrated tool suite designed for comprehensive analysis and functional interpretation of miRNAs, was utilized to predict potential upstream miRNAs of KRT8 and KRT19. The miRNA–KTR8 or miRNA–KRT19 networks were directly displayed on the webpage and downloaded as pictures.

### UALCAN database analysis

The methylation levels of promoters of KRT8 and KRT19 were determined using UALCAN database, which is a user-friendly, interactive web resource for analyzing cancer transcriptome data [17]. *P*-value < 0.05 was considered as statistically significant.

### Statistical analysis

The results were presented as mean ± SD. Unpaired Student’s *t*-test was employed to analyze the differences between two groups. Statistical analysis was performed using GraphPad Prism (version 7) and statistical significance was stated as two tailed *P*-value < 0.05.

## Results

### Screening of significant DEGs in LUAD

**Figure 1:**
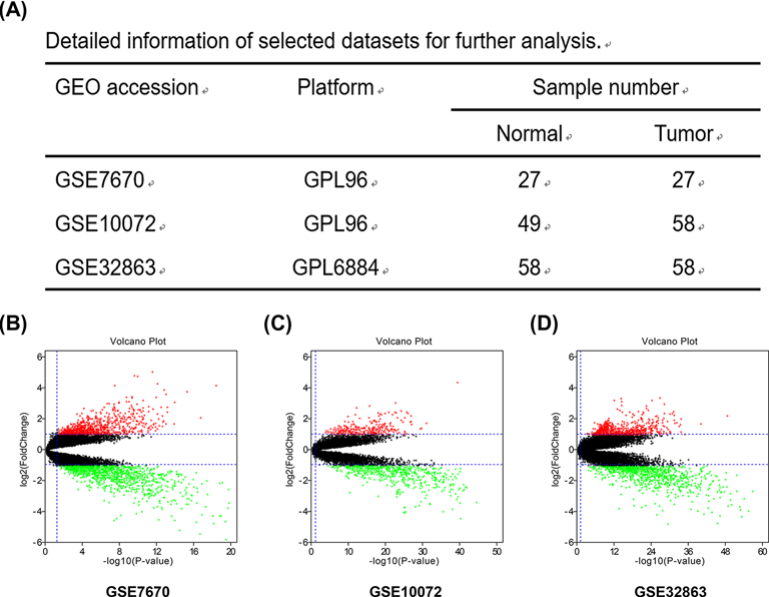
Volcano plots of differentially expressed genes (DEGs) from there datasets (GSE7670, GSE10072 and GSE32863) (**A**) The detailed information of three selected datasets for differential expression analysis. (**B**) DEGs identified in GSE7670 dataset. (**C**) DEGs identified in GSE10072. (**D**) DEGs identified in GSE32863. Note: These volcano plots showed all of the DEGs. The black dots represent genes that are not differentially expressed between lung adenocarcinoma tissues and normal lung tissues, and the green dots and red dots represent the down-regulated and up-regulated genes in cancer samples, respectively. Adj. *P*-value < 0.05 and |log_2_FC| > 1 were set as the cut-off criteria.

**Figure 2:**
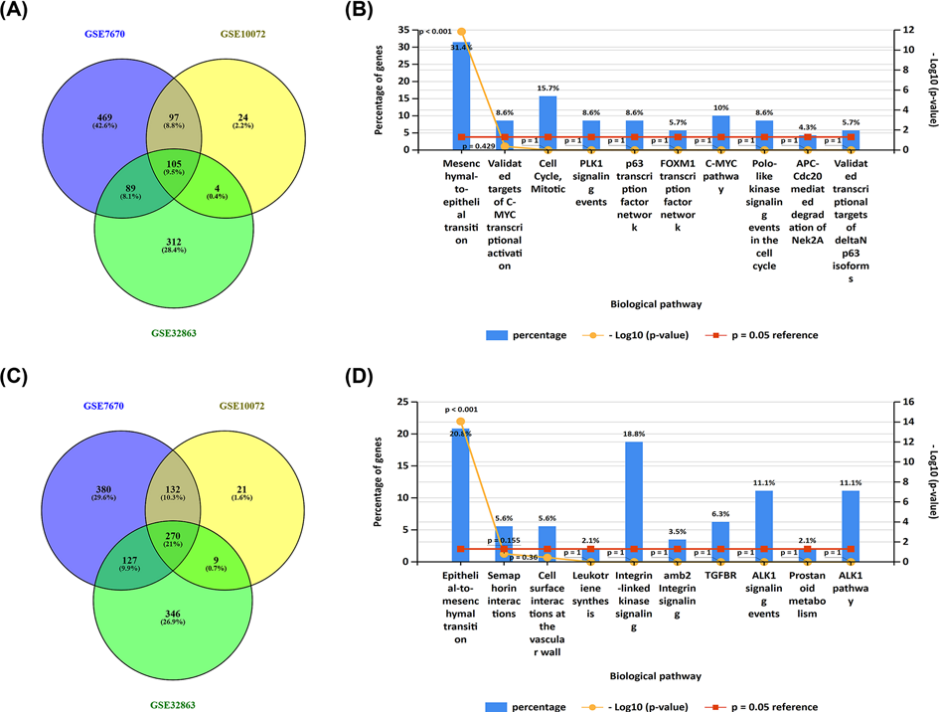
Pathway enrichment analysis for these DEGs commonly appeared in all three datasets (**A**) The intersection of up-regulated DEGs in all the three datasets. (**B**) Pathway enrichment analysis for these up-regulated DEGs that are commonly appeared in the three datasets using FunRich software. (**C**) The intersection of down-regulated DEGs in the three datasets. (**D**) Pathway enrichment analysis for these down-regulated DEGs that are commonly appeared in the three datasets using FunRich software.

Through a series of selection, three datasets (GSE7670, GSE10072 and GSE32863) were included for subsequent analyses. The detailed information of selected datasets was provided in Figure 1A. To identify the DEGs, we employed the online analytic tool GEO2R with the following parameters: adj. *P*-value < 0.05 and |log_2_FC| > 1. GEO2R analysis for GSE7670 found out 2048 DEGs, in which 913 genes were up-regulated and 1135 genes were down-regulated (Figure 1B); GEO2R analysis for GSE10072 found out 855 DEGs, in which 296 genes were up-regulated and 559 genes were down-regulated (Figure 1C); GEO2R analysis for GSE32863 found out 1428 DEGs, in which 550 genes were up-regulated and 878 genes were down-regulated (Figure 1D). The analytic results were showed in Supplementary Table S1. Next, these DEGs from three datasets were intersected to further screen DEGs, broadly dividing into two sets: up-regulated DEGs (Figure 2A) and down-regulated DEGs (Figure 2C). Eventually, a total of 105 up-regulated DEGs (Supplementary Table S2) and 270 down-regulated DEGs (Supplementary Table S3) were commonly appeared in all the three datasets. These DEGs were considered as significant DEGs, which were selected for following analyses.

### Pathway enrichment analysis for significant DEGs

In an attempt to study the potential functional roles and reveal the significantly altered signaling pathways in LUAD, we performed pathway enrichment analysis for the up-regulated significant DEGs and the down-regulated significant DEGs using FunRich software. The results showed that the upregulated significant DEGs were appeared in numerous biological pathways. The top 10 enriched pathways of the upregulated significant DEGs were presented in Figure 2B. The most enriched pathway was mesenchymal-to-epithelial transition (MET). Similarly, the down-regulated significant DEGs were also enriched in a variety of biological pathways (Figure 2D), among which epithelial-to-mesenchymal transition (EMT) was the most enriched pathway.

### Construction and analysis of PPI network

**Figure 3:**
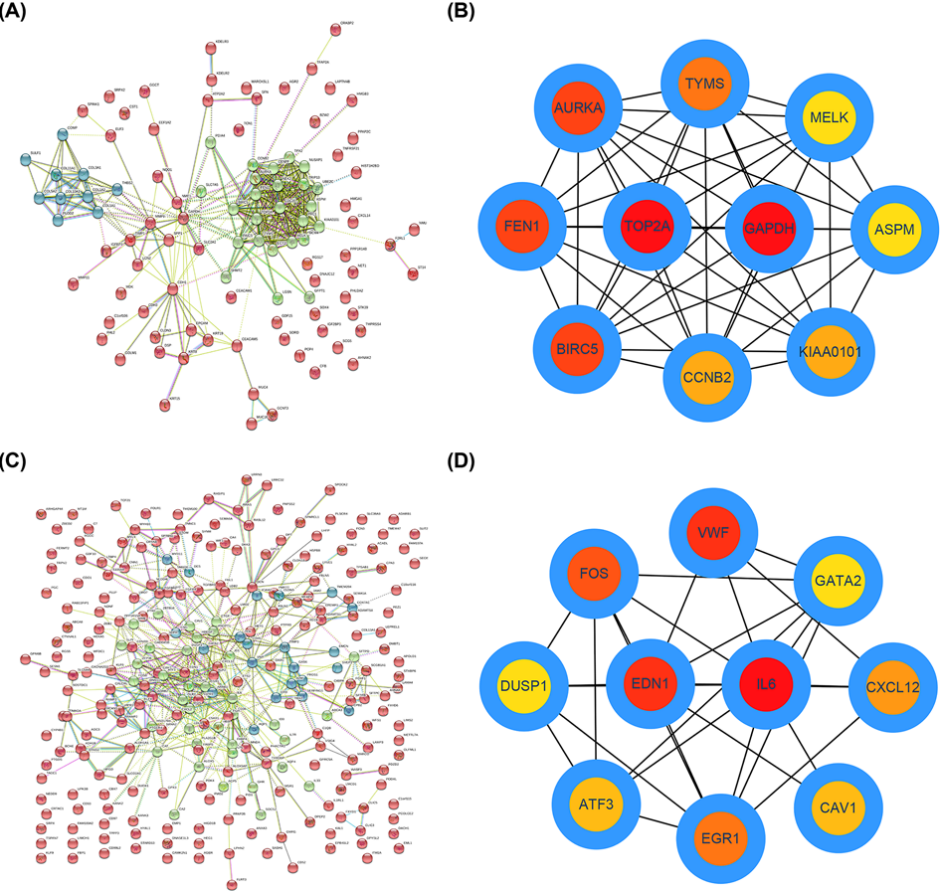
Identification of hub genes in the protein-protein interaction (PPI) networks of upregulated and downregulated significant DEGs (**A**) Establishment of PPI network of the up-regulated DEGs using STRING database. (**B**) The top 10 hub genes in the PPI network of the up-regulated DEGs according to node degree. (**C**) Establishment of PPI network of the down-regulated DEGs using STRING database. (**D**) The top 10 hub genes in the PPI network of the down-regulated DEGs according to node degree.

To investigate the association between the significant DEGs in LUAD, the online STRING database was first introduced to establish the PPI networks. Data from STRING database demonstrated that lots of these significant DEGs could interact with each other. The PPI network of the up-regulated significant DEGs was showed in Figure 3A, and the PPI network of the downregulated significant DEGs was presented in Figure 3C. In a PPI network, a gene with the more edges usually plays the more important role [18]. Therefore, we screened out hub genes according to the node degree calculated by CytoHubba of Cytoscape software. The top 50 hub genes and top 10 hub genes are shown in Table 1 and Figure 3B,D. Among these genes, GAPDH and IL6 revealed the highest node degrees, which were 26 and 49, respectively.

**Table 1:** The top 50 hub genes identified in the PPI networks

Up-regulated genes		Down-regulated genes	
Gene symbol	Degree	Gene symbol	Degree
GAPDH	26	IL6	49
TOP2A	26	VWF	30
FEN1	22	EDN1	30
BIRC5	22	FOS	22
AURKA	22	EGR1	21
TYMS	21	CXCL12	19
KIAA0101	20	ATF3	15
CCNB2	20	CAV1	15
TRIP13	19	CDH5	14
MELK	19	DCN	14
ASPM	19	GNG11	14
MCM4	19	DUSP1	14
CENPF	19	GATA2	14
NEK2	19	ANGPT1	14
UBE2C	19	ENG	13
ECT2	18	PTGER4	13
NUSAP1	18	TIMP3	13
CDH1	18	CD36	13
PRC1	18	CAT	12
CDC20	18	LDLR	12
TPX2	18	CTGF	12
PAICS	16	A2M	12
COL1A1	13	ADRB2	12
MMP9	13	TEK	12
COL3A1	12	KLF2	11
COL1A2	12	TGFBR2	11
SPP1	11	HBEGF	10
TIMP1	11	TYROBP	10
COL5A2	9	HSD17B6	10
COMP	8	ZFP36	10
COL11A1	8	CXCL2	10
KRT8	7	PLA2G1B	10
THBS2	7	LPL	10
NME1	7	ARRB1	10
COL10A1	7	KLF4	9
CEACAM5	6	CFD	9
EPCAM	6	DES	9
PLOD2	6	SERPING1	9
SLC2A1	5	PROS1	9
KRT19	5	FOSB	9
PDIA4	4	ALOX5	9
IGFBP3	4	EDNRB	9
LCN2	4	KLF6	8
SHMT2	4	PPP1R15A	8
ELF3	3	FGR	8
SULF1	3	ID1	8
NQO1	3	MYH11	8
F2RL1	3	SFTPD	8
MUC4	3	RAMP3	8
TFAP2A	2	CALCRL	8

### Identification of key genes-related to EMT/MET in LUAD

**Figure 4:**
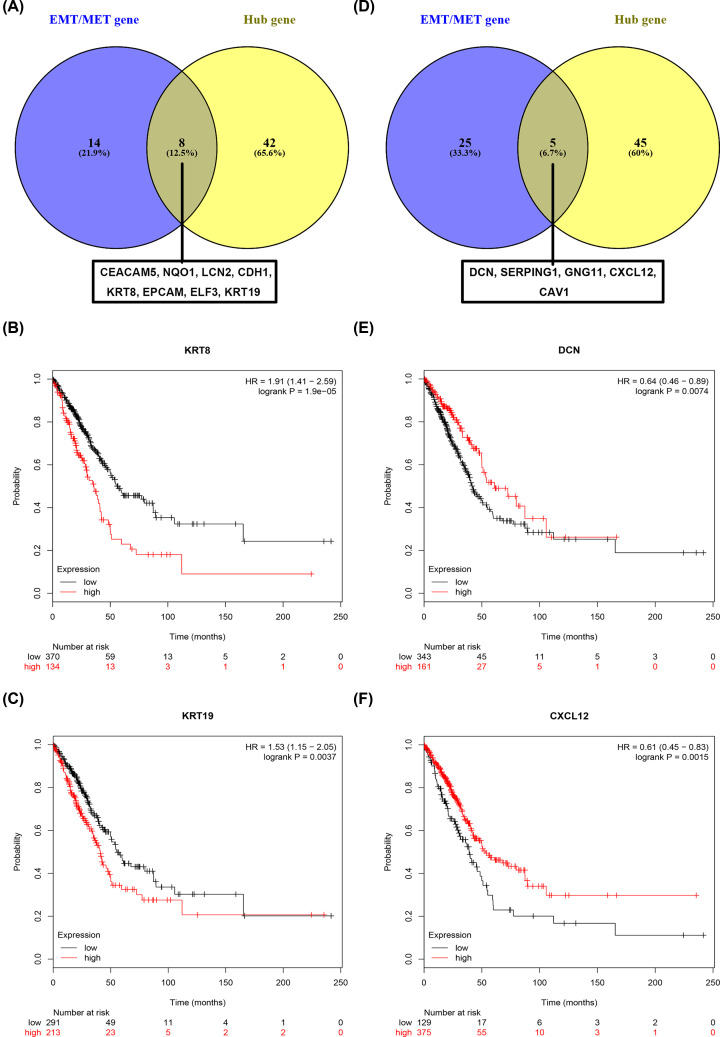
Screening of key genes in LUAD (**A**) Up-regulated genes (CEACAM5, NQO1, LCN2, CDH1, KRT8, EPCAM, ELF3 and KRT19) that are commonly appeared in EMT/MET pathway and the top 50 hub genes. (**B**) The prognostic value of KRT8 in patients with LUAD. (**C**) The prognostic value of KRT19 in patients with LUAD. (**D**) Down-regulated genes (DCN, SERPING1, GNG11, CXCL12 and CAV1) that are commonly appeared in EMT/MET pathway and the top 50 hub genes. (**E**) The prognostic value of DCN in patients with LUAD. (**F**) The prognostic value of CXCL12 in patients with LUAD.

**Figure 5:**
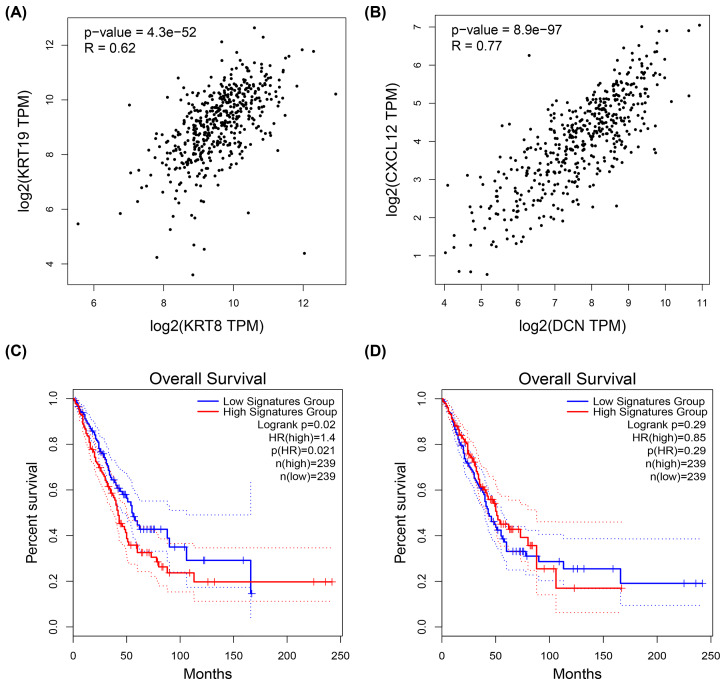
The correlation and joint prognostic values of KRT8–KRT19 and DCN–CXCL12 pairs in LUAD (**A**) The association between KRT8 expression and KRT19 expression in LUAD. (**B**) The association between DCN expression and CXCL12 expression in LUAD. (**C**) The joint prognostic value of KRT8–KRT19 pair in LUAD. (**D**) The joint prognostic value of DCN–CXCL12 pair in LUAD. *P*-value < 0.05 or logrank *P*-value < 0.05 was considered as statistically significant.

Previous analysis revealed that the up-regulated significant DEGs and the down-regulated significant DEGs were only markedly enriched in MET and EMT, respectively (Supplementary Table S4). Besides, the top 50 hub genes were also identified. In this part, we found out those genes that were commonly appeared in EMT/MET pathway gene set and the top 50 genes set (Figure 4A,D). Among the up-regulated significant DEGs and the down-regulated significant DEGs, 8 genes (CEACAM5, NQO1, LCN2, CDH1, KRT8, EPCAM, ELF3 and KRT19) and 5 genes (DCN, SERPING1, GNG11, CXCL12 and CAV1) were preliminarily identified as the key genes in LUAD, respectively. TCGA is an online platform for integrated analysis of molecular cancer data sets, which can provide an immeasurable source of knowledge in cancer research [19]. Therefore, TCGA expression and survival data of LUAD was utilized to validate the eight up-regulated key genes and five down-regulated key genes. We found that expression levels of eight up-regulated key genes were significantly increased (Supplementary Figure S1) and expression levels of five down-regulated key genes were markedly decreased (Supplementary Figure S2) in LUAD samples when compared with normal lung samples. These findings were completely identical with our previous analytic results. Subsequently, the prognostic values of eight up-regulated genes and five down-regulated genes were predicted and assessed using online Kaplan–Meier Plotter against the LUAD database. The results demonstrated that, among the eight up-regulated key genes, only KRT8 (Figure 4B) and KRT19 (Figure 4C) possessed significant unfavorable prognostic roles in patients with LUAD. Besides, we observed that, among the five down-regulated key genes, only LUAD patients with high expression of DCN (Figure 4E) and CXCL12 (Figure 4F) had favorable prognosis. Therefore, the four genes (KRT8, KRT19, DCN and CXCL12) were selected for further analysis. The correlation of KRT8 and KRT19 or DCN and CXCL12 expression were also assessed using GEPIA database. As shown in Figure 5A, KRT expression was positively associated with KRT19 expression in LUAD. Similarly, a positive correlation of DCN expression with CXCL12 expression was also observed (Figure 5B). As the strong linkage of KRT8–KRT19 or DCN– CXCL12 pair, the joint prediction capability of the two pairs for prognosis of LUAD were also evaluated. The result demonstrated that only overexpression of KRT8 and KRT19 could jointly indicated poor prognosis in LUAD (Figure 5C,D). Taken together, KRT8 and KRT19 were identified as two key genes-related to EMT/MET in LUAD.

### Exploration of two potential mechanisms of KRT8 and KRT19 in LUAD

**Figure 6:**
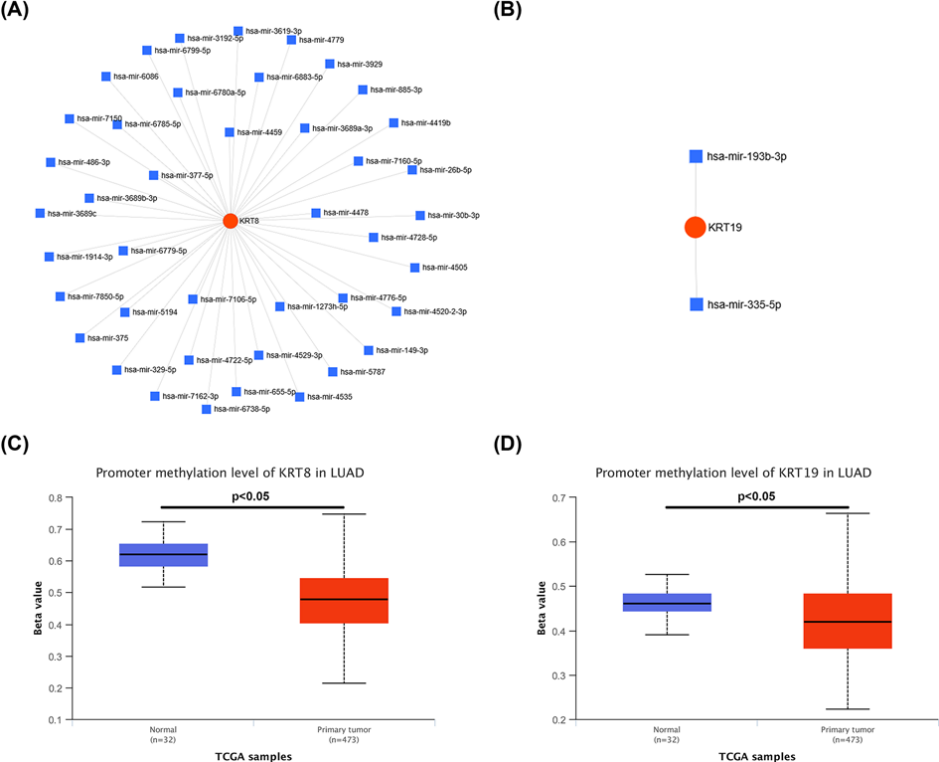
The potential mechanisms of KRT8 and KRT19 in LUAD (**A**) The potential miRNAs-KRT8 network constructed using miRNet database. (**B**) The potential miRNAs-KRT19 network constructed using miRNet database. (**C**) The methylation level of promoter of KRT8 in LUAD determined by UALCAN database. (**D**) The methylation level of promoter of KRT19 in LUAD determined by UALCAN database. *P*<0.05 was considered as statistically significant.

Next, we intended to explore the potential mechanisms of KRT8 and KRT19 in LUAD. As is known to all, gene expression is negatively regulated by microRNA (miRNA)12,13. Therefore, the potential upstream miRNAs of KRT8 and KRT19 were predicted using an integrated online tool, miRNet. Finally, a total of 42 and 2 potential miRNAs of KRT8 and KRT19 were identified, respectively. For better visualization, miRNAs-KRT8 and miRNAs-KRT19 networks were constructed as presented in Figure 6A,B, respectively. Methylation status of gene promoter partially accounts for gene dysregulation. Thus, we investigated the methylation levels of promoters of KRT8 and KRT19 in LUAD using UALCAN database. As shown in Figure 6C,D, both KRT8 and KRT19 were significantly hypomethylated in LUAD tissues when compared with normal lung tissues. All these findings suggest that aberrant regulation of KRT8 and KRT8 may result from dysregulation of upstream miRNAs and methylation status of gene promoter. Of course, more lab experiments need to be conducted to validate these results.

## Discussion

Lung cancer is the most prevalent cancer and the leading cause of cancer-related death worldwide [1]. LUAD, the most common histological type of lung cancer, which morbidity has been increasing year by year [20]. In this present study, we have comprehensively analyzed three gene expression microarray datasets of LUAD from the GEO database based on a series of bioinformatic analyses and partial experimental validation.

A total of 375 significant DEGs were identified, among which 105 genes were up-regulated and 270 genes were down-regulated. Pathway enrichment analysis demonstrated that 105 up-regulated genes and 270 down-regulated genes were significantly enriched in MET and EMT, respectively. MET and EMT are two reversible biological processes that involve the transition between mesenchymal-like state and epithelial-like state [21]. Extensive studies have reported that EMT is associated with multiple cancer characteristics, including metastasis [22,23] and chemoresistance [24]. Previous studies have also well documented that MET allows cancerous cells to regain epithelial properties and integrate into distant organs, thereby participating in the establishment and stabilization of distant metastases [25].

PPI networks among the up-regulated and down-regulated significant DEGs were subsequently constructed to explore associations between DEGs. With the help of Cytoscape software, we successfully identified the top 50 hub genes in the PPI networks according to node degree. Further analysis revealed that there were 8 up-regulated genes that were commonly appeared in the MET pathway and the top 50 hub genes, and 5 down-regulated genes that were also commonly appeared in the EMT pathway and the top 50 hub genes. The following analyses were mainly about the 8 up-regulated genes and 5 down-regulated genes, which were defined as the key genes-related to EMT/MET in LUAD. Expression of the 13 key genes were further validated and prognostic values of them in LUAD were also evaluated using TCGA LUAD expression and survival data. Identical with previous analytic results, all of these key genes were significantly dysregulated in LUAD. However, only KRT8, KRT19, DCN and CXCL12 had significant prognostic values. We also found that KRT8 (DCN) expression was positively linked to KRT19 (CXCL12) expression. Finally, the expression levels of the four genes were determined in cell lines by the method of qRT-PCR, and a similar outcome was observed.

Taken together, two up-regulated genes, KRT8 and KRT19, and two down-regulated genes, DCN and CXCL12, seem the most important genes in tumorigenesis and progression of LUAD. KRT8 and KRT19 are two members of the keratin family. Both KRT8 and KRT19 have been well demonstrated to function as two oncogenes in development of human cancers. For example, Tan et al*.* have suggested that KRT8 up-regulation promotes metastasis of clear cell renal cell carcinoma [26]. Fang et al*.* have found that KRT8 can facilitate gastric cancer progression and metastasis [27]. KRT19 acts as a key molecule in hepatocellular carcinoma invasion and angiogenesis [28]. KRT19 expression also correlates with poor prognosis in patients with breast cancer [29]. Furthermore, Wikman and co-workers have suggested that KRT8 and KRT19 are significantly up-regulated in LUAD tissues [30]. DCN encodes a proteoglycan named decorin. Multiple studies have suggested that DCN can suppress lung cancer progression. The group of Biaoxue R found that decreased expression of DCN correlates with lymphatic metastasis in patients with lung cancer [31]. Liang et al*.* showed that overexpression of DCN markedly inhibited proliferation and migration of LUAD A549 cell line [32]. Shi et al*.* suggested that DCN was responsible for progression of non-small-cell lung cancer by promoting cell proliferation and metastasis [33]. CXCL12 is also found to be closely associated with onset and development of human cancer [34]. These previous reports together with our current results indicate that the four key genes may play important roles in EMT/MET of LUAD.

Further investigation revealed that high expression of KRT8 and KRT19 could jointly forecast poor prognosis in LUAD. And the potential mechanisms of them in LUAD were preliminarily investigated. Numerous studies have validated that gene expression is negatively regulated by upstream miRNAs [12,13]. With the help of miRNet database, 42 and 2 potential miRNAs were predicted to target KRT8 and KRT19, respectively. Some of these miRNAs have been found to act as tumor suppressors in lung cancer. For example, miR-26b-5p suppresses proliferation, migration and invasion of lung cancer [35,36]. miR-335-5p inhibits proliferation of lung cancer by targeting Tra2 [37]. Dysregulated methylation status of gene promoter can lead to gene aberrant expression [38,39]. Therefore, we further detected the methylation levels of two up-regulated genes, KRT8 and KRT19. Intriguingly, both promoters of KRT8 and KRT19 were significantly hypomethylated in LUAD tissues compared with normal controls.

Although excellent results are obtained, lacking of experimental confirmation is an obvious limitation in this study. In the future, much more lab experiments and large-scale clinical trials need to be conducted to further validate these findings.

## Conclusions

On the whole, all current findings confirmed that KRT8 and KRT19 are two key genes in the pathogenesis of LUAD. Both of them are significantly hypomethylated and overexpressed in LUAD and linked to unfavorable prognosis. KRT8 and KRT19 may be utilized as two diagnostic, therapeutic and prognostic biomarkers in LUAD in the future.

## Supplementary Material

Supplementary Figures S1-S2 and Tables S1-S4

## Abbreviations

EMT: epithelial-to-mesenchymal transition
GEO: Gene Expression Omnibus
GEPIA: Gene Expression Profiling Interactive Analysis
GTEx: Genotype-Tissue Expression
LUAD: lung adenocarcinoma
MET: mesenchymal-to-epithelial transition
PPI: protein–protein interaction
qRT-PCR: quantitative real-time PCR
STRING: Search Tool for the Retrieval of Interacting Genes
TCGA: The Cancer Genome Atlas
