# Supplementary material for: KRT8 and KRT19, associated with EMT, are hypomethylated and overexpressed in lung adenocarcinoma and link to unfavorable prognosis
Source: Biosci Rep. 2020 Jul 3;40(7):BSR20193468. doi: 10.1042/BSR20193468 (PMC7335829; doi:10.1042/BSR20193468)
Supplement: Supplementary Figures S1-S2 and Tables S1-S4 [file BSR-2019-3468_supp.pdf]

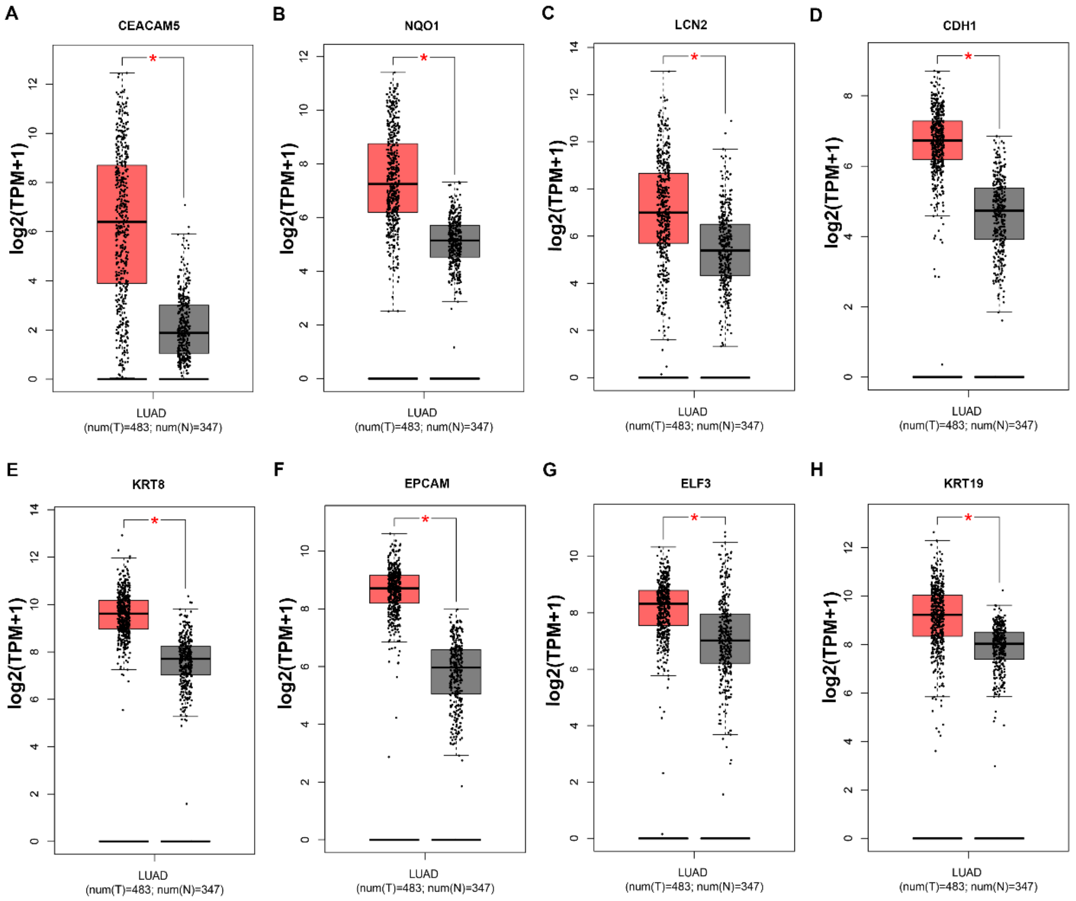

Figure S1. The expression of potential upregulated genes in LUADdetermined by GEPIA database. (A) CEACAM5 expression; (B) NQO1 expression; (C) LCN2 expression; (D) CDH1 expression; (E) KRT8 expression; (F) EPCAM expression; (G) ELF3 expression; (H) KRT19 expression. Note: red column represents tumor group and gray column represents normal group. *P<0.05.

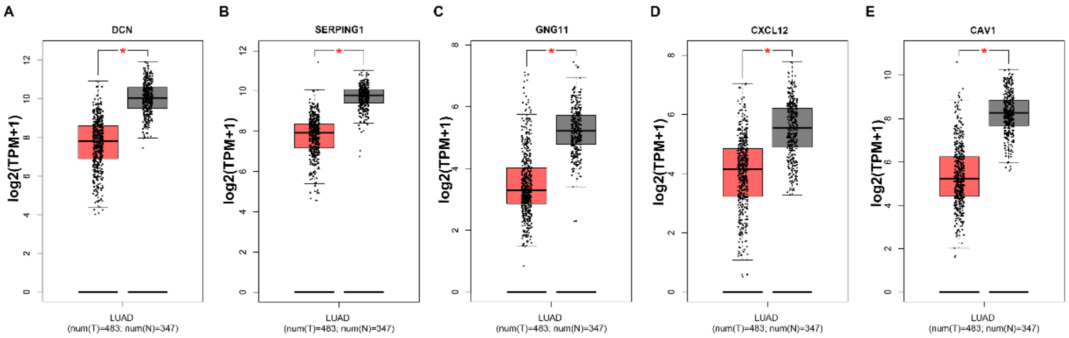

Figure S2. The expression of potential downregulated genes in LUAD determined by GEPIA database. (A) DCN expression; (B) SERPING1 expression; (C) GNG11 expression; (D) CXCL12 expression; (E) CAV1 expression. Note: red column represents tumor group and gray column represents normal group. *P<0.05.

Table S1. Numbers of differentially expressed genes between LUAD tissues and normal lung tissues from three datasets, namely GSE7670, GSE10072 and GSE32863.

GEO accession	Upregulated gene count	Downregulated gene count	Total
GSE7670	913	1135	2048
GSE10072	296	559	855
GSE32863	550	878	1428

Table S2. Upregulated genes commonly appeared in all three datasets (GSE7670, GSE10072 and GSE32863).

Upregulated genes
COL11A1
CDH3
SPINK1
SPP1
COL10A1
TMPRSS4
TOP2A
NEK2
CRABP2
COL1A1
COMP
NMU
EEF1A2
SFN
MMP11
BIRC5
CEACAM5
CST1
GOLM1
CDC20
GCNT3
MMP12
KRT15
LGSN
ASPM
MELK
CXCL14
CLDN3
CD24
CENPF
MCM4
COL1A2
PCP4
COL3A1
PHLDA2
PLPP2
TPX2

HMGB3
TFAP2A
SULF1
SOX4
KIAA0101
THBS2
AGR2
IGF2BP3
GDF15
MMP9
AURKA
MDK
NQO1
LCN2
NUSAP1
MUC16
KDELR2
CCNB2
UBE2C
DNAJC12
GGCT
BZW2
SLC2A1
TYMS
ST14
SCG5
NME1
IGFBP3
COL5A2
TCN1
PAICS
DSP
RGS17
HIST1H2BD
KDELR3
CEACAM1
SLC7A5
F2RL1
SRPX2
TNFRSF21

STK39
PRC1
PPP1R14B
LAPTM4B
FHL2
PLOD2
NET1
HMGA1
SHMT2
SORD
MARCKSL1
C1orf106
FEN1
TIMP1
CDH1
PDIA4
MUC4
KRT8
TRIP13
AHNAK2
EPCAM
GAPDH
GFPT1
ECT2
ATP2A2
ELF3
CFB
KRT19

Table S3. Downregulated genes commonly appeared in all three datasets (GSE7670, GSE10072 and GSE32863).

Downregulated genes
FLRT3
FBP1
LAMA4
PODXL
CRTAC1
SEMA5A
SPTBN1
MAOA
TPSAB1
TIMP3
RGS5
EPB41L2
EGR1
GADD45B
FBLN1
DUOX1
HPGD
TRPV2
LMCD1
CD93
HYAL2
SESN1
AQP1
STOM
ACP5
C1QB
SLC39A8
RCAN1
C5AR1
IL7R
GRK5
MYH11
PHACTR2
DMBT1
SPOCK2
WFS1

ENG
CTNNAL1
PLPP3
TYROBP
FERMT2
RAB11FIP1
GATA2
GAS6
EPAS1
WASF3
SFTPB
EML1
HEY1
TMEM47
FABP5
PID1
FOLR1
SLPI
PAPSS2
SMAD6
DCN
TBX2
SECISBP2L
CPA3
ARRB1
CAT
ZFP36
RASL12
SASH1
DUSP1
LDLR
ALOX5
CLIC5
C4BPA
KANK2
ALOX5AP
MSR1
GPM6B
PTRF
A2M

DKK3
PROS1
EMP1
GPRC5A
NDNF
HEG1
DNASE1L3
SLCO2A1
EFEMP1
OLFML1
PPP1R15A
CBX7
GMFG
TGFBR2
ADGRE5
PLSCR4
OLR1
ADAMTS8
SLIT2
SYNM
ATF3
PLLP
ZEB2
FOS
NEDD9
APOLD1
TACC1
ETS2
TMEM204
FAM189A2
SERPING1
KLF6
STARD13
MNDA
GHR
ADRB2
ICAM2
DPT
FEZ1
LMO2

JAM2
FXYD6
S100A4
COX7A1
ID1
HBEGF
FGR
RRAS
PTGDS
ARHGAP44
HOXA5
CAMK2N1
COL13A1
KLF9
LMO7
VSIG4
CXCL2
GNG11
ANXA3
DPYSL2
METTL7A
HSPB8
CTGF
ITM2A
NR4A2
PMP22
CXCL12
EDN1
CNN1
PDK4
AQP4
MYL9
SPARCL1
CDO1
LHFP
CA2
MYH10
SEMA6A
DACH1
C1orf115

SDPR
CALCRL
SRPX
PCOLCE2
HSD17B6
ABCA3
KLF2
LIMS2
SOCS2
LIMCH1
P3H2
PECAM1
PLA2G1B
ID3
LRRN3
CRYAB
RASIP1
CD52
GPX3
IL6
PTGER4
ZBED2
ADGRL2
PTPRB
CX3CR1
TSPAN7
LAMP3
CLIC3
FBLN5
VWF
FMO2
FOXF1
ACADL
CACNA2D2
S1PR1
GPC3
CAV2
PGC
AHNAK
ADARB1

TGFBR3
CPB2
LPL
DES
ZBTB16
CLDN5
LRRC32
ANGPT1
PDZD2
GDF10
BCHE
TBX3
LYVE1
RAMP3
ALDH1A1
KLF4
RECK
FHL1
EDNRB
ADAMTS1
LTBP4
C7
SFTPC
CLDN18
VIPR1
NEDD4L
CDH5
TEK
WFDC1
TPPP3
MME
FGFR4
UPK3B
SVEP1
AOC3
ACKR1
HYAL1
CFD
CD36
FXYD1

ANOS1
FAM107A
RGCC
ADH1B
IL33
LDB2
EMCN
DPEP2
HBB
CAV1
SFTPD
KANK3
SLC6A4
MARCO
ABCA8
SCGB1A1
IL1RL1
TCF21
CYP4B1
CA4
MFAP4
HIGD1B
RAMP2
STXBP6
ADIRF
MT1M
FOSB
FCN3
TMEM100
FABP4
WIF1
SOSTDC1
AGER
TNNC1

Table S4. Upregulated genes enriched in mesenchymal-to-epithelial transition (MET) and downregulated genes enriched in epithelial-to-mesenchymal transition (EMT).

Upregulated genes in MET	Downregulated genes in EMT
CDH3	FBLN1
TMPRSS4	FERMT2
SFN	DCN
CEACAM5	GPM6B
KRT15	PTRF
CLDN3	HEG1
CD24	EFEMP1
PHLDA2	SLIT2
AGR2	SYNM
GDF15	ZEB2
NQO1	SERPING1
LCN2	DPT
ST14	JAM2
DSP	FXYD6
CEACAM1	PTGDS
SORD	VSIG4
C1orf106	GNG11
CDH1	ITM2A
KRT8	PMP22
EPCAM	CXCL12
ELF3	MYL9
KRT19	SPARCL1
	LHFP
	MYH10
	SRPX
	CRYAB
	RECK
	FHL1
	CAV1
	MFAP4
